# Central Sensitization as a Marker of Cognitive and Emotional Vulnerability in Chronic Low Back Pain

**DOI:** 10.3390/brainsci16030290

**Published:** 2026-03-05

**Authors:** Anna Anselmo, Irene Cappadona, Maria Pagano, Alice Laudisio, Rosaria De Luca, Fabrizio Russo, Giulia Martello, Davide Cardile, Angelo Quartarone, Rocco Salvatore Calabrò, Francesco Corallo

**Affiliations:** 1 IRCCS Centro Neurolesi Bonino-Pulejo, Via Palermo, S.S. 113, C. da Casazza, 98124 Messina, Italy; anna.anselmo@irccsme.it (A.A.);; 2 Operative Research Unit of Orthopaedic and Trauma Surgery, Fondazione Policlinico Universitario Campus Bio-Medico, Via Alvaro del Portillo 200, 00128 Rome, Italy; 3 Research Unit of Orthopaedic and Trauma Surgery, Departmental Faculty of Medicine and Surgery, Università Campus Bio-Medico di Roma, Via Alvaro del Portillo 21, 00128 Rome, Italy

**Keywords:** central sensitization, chronic low back pain, pain catastrophizing, depression, cognitive functioning

## Abstract

**Highlights:**

**What are the main findings?**

- Central sensitization in chronic low back pain is associated with worse cognitive performance, higher depressive symptoms, and increased pain catastrophizing across all domains.
- Patients with central sensitization represent a distinct clinical subgroup characterized by a multidimensional vulnerability profile extending beyond pain intensity.

**What are the implications of the main findings?**

- The Central Sensitization Inventory may be a valuable tool for early identification and clinical stratification of patients at risk for cognitive and emotional dysfunction.
- Integrating central sensitization assessment into routine practice could support personalized, multimodal interventions targeting cognitive, emotional, and behavioral processes.

**Abstract:**

**Background and Aim**: Low back pain (LBP) represents an important public health issue, with approximately 20% of acute cases progressing to chronic low back pain (CLBP). In addition to pain, patients with CLBP also suffer from reduced cognitive performance, depressive symptoms and catastrophic thoughts. Central sensitization (CS) is considered a key point in pain persistence. This study examines CS and its impact on cognitive, emotional, and behavioral functioning in patients with CLBP. **Methods**: In this cross-sectional study, 67 patients with CLBP were classified using the Central Sensitization Inventory (CSI) into groups with (WCS, *n* = 32) and without central sensitization (WoCS, *n* = 35). Cognitive functioning was assessed using the Montreal Cognitive Assessment (MoCA), emotional functioning using the Center for Epidemiologic Studies Depression Scale (CES-D), and behavioral functioning using the Pain Catastrophizing Scale (PCS), including helplessness, rumination, and magnification domains. Normality was assessed using the Shapiro–Wilk test. Between-group comparisons were performed using Mann–Whitney U, chi-square, or Welch’s *t*-tests. Multivariable linear regression analyses adjusted for age and gender were conducted. **Results**: Compared with the WoCS group, patients with central sensitization were older (median 58 vs. 50 years, *p* = 0.001) and more frequently female (71.9% vs. 40.0%, *p* = 0.018). The WCS group showed higher PCS total scores (31.8 ± 14.2 vs. 16.0 ± 11.9), higher helplessness (14.3 ± 6.1 vs. 6.9 ± 5.5), rumination (12.7 ± 6.2 vs. 7.0 ± 4.8), and magnification scores (4.8 ± 2.4 vs. 2.1 ± 2.1), higher CES-D scores (26.3 ± 10.4 vs. 11.7 ± 7.2), and lower MoCA scores (23.6 ± 3.0 vs. 26.1 ± 2.1) (all *p* < 0.001). All associations remained significant after adjustment for age and gender. **Conclusions:** Central sensitization in CLBP is independently associated with greater pain catastrophizing across all domains, increased depressive symptoms, and reduced cognitive performance, supporting its role as a multidimensional clinical phenotype.

## 1. Introduction

Chronic low back pain (CLBP) represents one of the leading causes of disability worldwide, exerting a substantial impact not only on physical functioning but also on cognitive, emotional, and behavioral dimensions [1]. Despite advances in diagnostic procedures and therapeutic strategies, approximately one fifth of patients progress to a chronic condition, characterized by persistent pain, reduced quality of life, and limited responsiveness to conventional treatments [2]. This scenario has progressively challenged the traditional biomechanical model, fostering the emergence of more complex and integrative interpretative approaches. Within this framework, central sensitization (CS) is currently regarded as a key mechanism underlying the persistence of chronic pain [3]. CS refers to a state of hyperexcitability of the central nervous system, in which circuits involved in sensory and nociceptive processing become dysregulated, leading to amplification and persistence of pain signals independently of ongoing peripheral tissue damage [4]. However, despite increasing scientific interest, CS remains a clinical construct that is often underrecognized and underutilized in routine clinical practice. One of the most widely used instruments for identifying central sensitization is the Central Sensitization Inventory (CSI), a self-report questionnaire developed to assess symptoms related to altered central pain processing [5]. The CSI has been validated in patients with chronic low back pain, and recent evidence using machine-learning approaches has established clinically relevant cut-off values with good discriminative accuracy for identifying central sensitization in this population [6]. The CSI captures the multidimensional nature of CS by encompassing not only somatic symptoms but also cognitive and emotional manifestations frequently reported by patients with chronic pain. Despite its growing validation in both clinical and research settings, the CSI is still infrequently integrated into CLBP assessment pathways, and its predictive value with respect to neuropsychological functioning remains largely unexplored [7]. Concurrently, accumulating evidence suggests that patients with CLBP frequently exhibit neuropsychological impairments across multiple domains. From a cognitive perspective, alterations in sustained and selective attention, working memory, and executive functions have been reported, with significant repercussions on planning abilities, problem-solving, and adaptation to environmental demands [8,9]. These cognitive deficits do not merely represent secondary consequences of persistent pain, but rather appear to reflect a dysfunctional competition for attentional resources and an altered modulation of frontoparietal networks involved in both pain processing and higher-order cognitive functions [10,11]. In addition to cognitive alterations, CLBP is commonly associated with depressive symptoms, which further exacerbate pain perception, disability, and treatment non-adherence [12]. Depression in the context of chronic pain should not be conceptualized solely as a psychological reaction to suffering, but rather as an integral component of a shared neurobiological dysregulation, in which mood regulation and nociceptive systems are closely interconnected [13,14]. In this context, central sensitization may represent a linking mechanism between persistent pain and emotional dysregulation [15]. Another key factor is pain catastrophizing, defined as a maladaptive cognitive style characterized by rumination, magnification of threat, and feelings of helplessness in relation to pain [16]. Pain catastrophizing is not only associated with greater pain intensity and disability but also appears to contribute to the maintenance of central sensitization through mechanisms of hypervigilance and enhanced attentional focus on pain-related stimuli [17]. From this perspective, cognition, emotion, and behavior do not constitute separate dimensions, but rather interdependent components of a dysfunctional network sustained by central sensitization, which contributes to pain chronicity and clinical complexity [18]. In light of these considerations, the present study aims to investigate the role of central sensitization, assessed through the Central Sensitization Inventory, in CLBP, and to examine its association with cognitive, emotional, and behavioral functioning. The overarching goal is to contribute to a more articulated and clinically meaningful understanding of CLBP, promoting the use of the CSI as a key tool for early identification of at-risk profiles and for the development of truly personalized multimodal interventions.

## 2. Methods

### 2.1. Study Design

The study adopted a cross-sectional observational design aimed at exploring the role of central sensitization and its association with cognitive, emotional, and behavioral functioning in patients with CLBP. The present analysis represents a cross-sectional baseline (T0) investigation nested within a larger prospective multi-time-point study [19]. The current manuscript focuses on exploratory analyses of preliminary neuropsychological correlations.

### 2.2. Participants

A total of 67 patients with chronic low back pain (CLBP) were recruited at the Fondazione Policlinico Universitario Campus Bio-Medico of Rome. Inclusion criteria were age between 18 and 65 years, clinically significant low back pain (score > 4 on the Numeric Rating Scale, NRS), and pain duration between 6 weeks and 12 months. Exclusion criteria included major neurological disorders, severe psychiatric conditions, or other clinical conditions that could interfere with neuropsychological assessment.

All participants underwent orthopedic clinical evaluation and lumbar MRI to confirm the diagnosis of CLBP and to exclude specific structural spinal pathologies. Years of education were recorded for all participants, as educational level is known to influence performance on cognitive screening measures.

The protocol of the entire project was approved by the Ethics Committee CET Lazio Area 2 (ID No. 181.24 CET2 CBM, approval date: 31 July 2024). All participants provided written informed consent prior to inclusion in the study, which was conducted in accordance with the principles of the Declaration of Helsinki.

### 2.3. Assessment of Central Sensitization

Central sensitization was assessed using the Central Sensitization Inventory (CSI). A CSI score ≥ 40 was used as a cut-off to identify the presence of central sensitization, in accordance with the literature [20]. Based on the total CSI score, participants were divided into two groups: patients with central sensitization (WCS) and patients without central sensitization (WoCS).

### 2.4. Neuropsychological Assessment

All participants underwent a standardized neuropsychological assessment. Global cognitive performance was evaluated using the Montreal Cognitive Assessment (MoCA). MoCA scores were adjusted for age and education using Italian normative data to minimize the influence of sociodemographic factors on global cognitive performance. Depressive symptoms were assessed with the Center for Epidemiologic Studies Depression Scale (CES-D), while pain-related catastrophizing was measured using the Pain Catastrophizing Scale (PCS).

In addition to neuropsychological measures, information regarding medication use and physical activity level was systematically recorded; however, these variables were not included in the primary analyses, as the study focused on cross-sectional associations between central sensitization and neuropsychological measures at baseline. A detailed description of the assessment instruments is provided in Table 1.

### 2.5. Statistical Analysis

All statistical analyses were conducted to examine the association between central sensitization and cognitive, emotional, and behavioral functioning in patients with chronic low back pain. Continuous variables were summarized using mean and standard deviation when normally distributed, and median with interquartile range (IQR) when non-normally distributed, while categorical variables were summarized using frequencies and percentages. The distribution of continuous variables was assessed using the Shapiro–Wilk test separately within each group. Age showed a non-normal distribution in the group with central sensitization and was therefore summarized as median and IQR and analyzed using non-parametric methods in unadjusted comparisons. Between-group differences in demographic variables were assessed using the Mann–Whitney U test for age and the chi-square test for gender distribution. Between-group comparisons for clinical and psychological outcomes (CSI, PCS total score, PCS domain scores, MoCA, and CES-D) were performed using Welch’s independent-samples *t*-tests to account for potential heterogeneity of variances. To evaluate whether central sensitization was independently associated with cognitive, emotional, and behavioral outcomes, multiple linear regression analyses were conducted. Separate models were fitted for PCS total score, PCS helplessness, rumination, magnification, MoCA, and CES-D as dependent variables. Group (with vs. without central sensitization) was entered as the main independent variable, while age and gender were included as covariates. Both unstandardized (B) and standardized (β) regression coefficients were examined. Unstandardized coefficients were reported to allow clinical interpretability, while standardized coefficients were used to compare the relative contribution of predictors measured on different scales. All statistical tests were two-tailed, and statistical significance was set at *p* < 0.05.

## 3. Results

Sixty-seven patients with chronic low back pain were included in the analysis, of whom 32 were classified as with central sensitization (WCS) and 35 as without central sensitization (WoCS). Patients in the WCS group were significantly older than those in the WoCS group (median 58 years [IQR 51–61] vs. 50 years [IQR 35–54]; *p* = 0.001) and included a higher proportion of females (71.9% vs. 40.0%; *p* = 0.018). As expected, CSI scores were significantly higher in the WCS group compared with the WoCS group (57.5 ± 11.9 vs. 21.9 ± 10.2; *p* < 0.001).

Cognitive functioning was significantly poorer in the WCS group, as reflected by lower MoCA scores compared with the WoCS group (23.6 ± 3.0 vs. 26.1 ± 2.1; *p* < 0.001). This difference remained significant after adjustment for age and gender, with belonging to the WoCS group associated with higher MoCA scores (B = 2.41, 95% CI 1.36–3.46; β = 0.41; *p* < 0.001). Emotional functioning also differed significantly between groups, with higher depressive symptom severity observed in patients with central sensitization, indicated by higher CES-D scores (26.3 ± 10.4 vs. 11.7 ± 7.2; *p* < 0.001). In adjusted analyses, group membership remained an independent predictor of depressive symptoms, with the WoCS group showing significantly lower CES-D scores (B = −14.62, 95% CI −18.7 to −10.5; β = −0.64; *p* < 0.001). Behavioral functioning differed markedly between groups. Patients with central sensitization reported substantially higher PCS total scores than those without central sensitization (31.8 ± 14.2 vs. 16.0 ± 11.9; *p* < 0.001). After adjustment for age and gender, central sensitization remained independently associated with higher pain catastrophizing, with the WoCS group showing a significantly lower PCS total score (B = −15.24, 95% CI −19.8 to −10.6; β = −0.52; *p* < 0.001).

Unadjusted between-group comparisons are summarized in Table 2.

**Figure 1:**
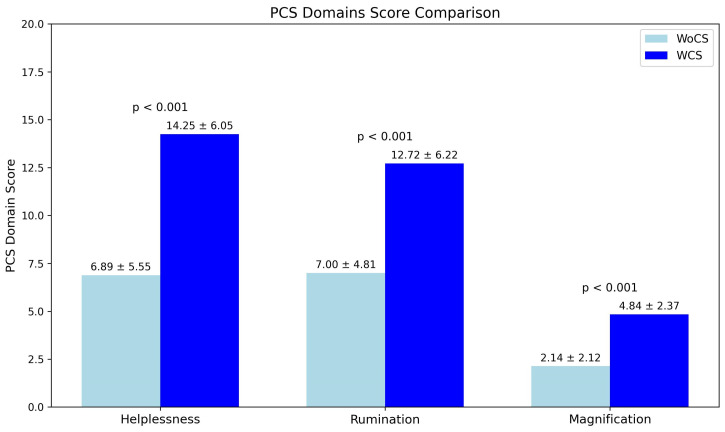
PCS Domain score comparison between groups.

Analysis of PCS domains demonstrated consistently higher scores in the WCS group. Specifically, helplessness scores were significantly higher in patients with central sensitization compared with those without central sensitization (14.3 ± 6.1 vs. 6.9 ± 5.5; *p* < 0.001). Similarly, rumination scores were higher in the WCS group (12.7 ± 6.2 vs. 7.0 ± 4.8; *p* < 0.001), as were magnification scores (4.8 ± 2.4 vs. 2.1 ± 2.1; *p* < 0.001) (Figure 1).

After adjustment for age and gender, central sensitization remained an independent predictor of all behavioral, cognitive, and emotional outcomes. Group differences in PCS total score, PCS domain scores (helplessness, rumination, and magnification), MoCA, and CES-D all remained statistically significant (all adjusted *p* < 0.001), indicating that the observed impairments in patients with central sensitization were not explained by demographic differences alone.

Detailed regression coefficients and confidence intervals for all adjusted associations are reported in Table 3.

## 4. Discussion

The present study investigated the association between central sensitization and cognitive, emotional, and behavioral functioning in patients with CLBP. Our findings indicate that patients presenting a profile compatible with central sensitization exhibit a more complex and severe clinical phenotype, characterized by poorer global cognitive performance, higher levels of pain catastrophizing, and more severe depressive symptoms compared to patients without central sensitization. These results support the view that central sensitization represents not only a mechanism of pain amplification, but also a multidimensional condition involving cognitive and emotional dysregulation that may contribute to pain persistence and disability [24,25].

### 4.1. Central Sensitization and Clinical Stratification

The adoption of a CSI cut-off score ≥ 40 to identify a clinically relevant profile of central sensitization is consistent with the previous literature proposing this threshold as a reliable discriminator between patients with and without central sensitization syndromes [26]. The clear separation observed between WCS and WoCS patients across multiple outcome domains reinforces the clinical utility of the CSI as a screening instrument in CLBP. Importantly, this stratification allowed the identification of a subgroup of patients characterized by a broader vulnerability profile, extending beyond pain intensity to include cognitive and emotional domains [27]. This interpretation should be considered in light of the multidimensional nature of the Central Sensitization Inventory. The CSI is designed to capture the clinical symptom profile associated with central sensitization rather than to provide a direct physiological measure of central nervous system sensitization. Some items refer to affective and cognitive experiences, such as mood changes, fatigue, and concentration difficulties; however, these aspects are represented by brief screening items and do not constitute a comprehensive evaluation comparable to dedicated mood or cognitive assessment tools. Instead, their inclusion reflects the biopsychosocial framework underlying central sensitization, in which sensory, emotional, and cognitive processes are intrinsically interconnected. From this perspective, the associations observed in our study support the view of central sensitization as a multidimensional clinical construct encompassing vulnerability across cognitive and emotional domains [6]. This supports the notion that central sensitization may represent a key construct for clinical stratification rather than a mere descriptive label.

### 4.2. Central Sensitization, Pain Catastrophizing, and Depressive Symptoms

The finding of significantly higher PCS scores in patients with central sensitization is in line with previous evidence indicating that symptoms of central sensitization are closely associated with maladaptive pain-related cognitions [28]. Pain catastrophizing, particularly its helplessness and rumination components, has been shown to promote hypervigilance toward bodily sensations and persistent attentional focus on pain, thereby facilitating the maintenance of central sensitization processes [29]. In this framework, catastrophizing may act both as a consequence and as a maintaining factor of sensitization, reinforcing pain-related neural amplification through top-down cognitive mechanisms [30]. Similarly, the presence of more severe depressive symptoms among patients with central sensitization is consistent with a substantial body of literature documenting the frequent co-occurrence of chronic pain and depression [31]. The CES-D was selected as a validated instrument for screening depressive symptomatology in chronic pain populations. Future studies may benefit from including additional measures to further characterize mental health profiles.

Rather than representing a simple psychological reaction to pain, depressive symptoms may share common neurobiological substrates with central sensitization, including altered monoaminergic modulation and dysfunction of cortico-limbic circuits involved in affective pain processing [32]. The positive association between CSI and CES-D scores observed in our sample supports the hypothesis that central sensitization constitutes a clinical nexus in which pain amplification and emotional dysregulation mutually reinforce each other, leading to greater clinical complexity [33].

### 4.3. Central Sensitization and Cognitive Functioning

With regard to cognitive assessment, the Italian version of the Montreal Cognitive Assessment (MoCA) was used, validated and calibrated for the Italian population (2006 version), which sets a normal cut-off score of 26. In line with the recommendations of the Italian validation, scores between 23 and 26 were interpreted with caution and always in the relevant clinical context [34]. For the purposes of analysis, only scores ≤ 24 were considered indicative of lower cognitive performance. All MoCA scores were also corrected for age and education level in order to reduce potential biases related to sociodemographic factors. In the present study, MoCA scores were not interpreted diagnostically, but as an overall measure of cognitive performance [35]. Therefore, the reduced performance observed in patients with central sensitization should be understood as an expression of increased cognitive load associated with chronic pain, rather than as indicative of primary neurodegenerative processes [36].

It should be noted that cognitive functioning was assessed using a global screening instrument rather than a comprehensive neuropsychological battery. Accordingly, our findings should be interpreted as reflecting differences in overall cognitive performance, rather than diagnostic evidence of specific cognitive impairment. This interpretation is consistent with evidence of a negative relationship between the severity of central sensitization and cognitive performance, and supports the hypothesis that central sensitization identifies, within the CLBP population, a subgroup that is particularly vulnerable to cognitive difficulties related to the interference of persistent pain on attentional and executive processes [37,38].

### 4.4. Integrated Interpretation and Clinical Implications

Taken together, these findings support a biopsychosocial model of CLBP in which central sensitization, cognitive vulnerability, emotional distress, and maladaptive pain-related cognitions converge to define a more severe and treatment-resistant clinical profile. The integration of CSI assessment into routine clinical evaluation may therefore enhance patient stratification and facilitate the identification of individuals who may benefit from tailored multimodal interventions [20]. From a clinical standpoint, early recognition of central sensitization could inform the implementation of interventions targeting not only peripheral or biomechanical factors, but also cognitive and emotional processes, such as pain neuroscience education, cognitive–behavioral strategies, and psychologically informed rehabilitation approaches [39,40]. Addressing catastrophizing and depressive symptoms may be particularly relevant in patients with central sensitization, as these factors appear to be closely intertwined with altered central pain processing and cognitive burden [41,42,43].

## 5. Strengths

This study presents several strengths. First, it adopts an integrated biopsychosocial approach to chronic low back pain, simultaneously investigating central sensitization, cognitive functioning, depressive symptoms, and pain catastrophizing. This multidimensional perspective allows for a more comprehensive understanding of CLBP beyond traditional biomechanical models. Second, the use of validated and widely employed instruments such as the Central Sensitization Inventory (CSI), Montreal Cognitive Assessment (MoCA), Center for Epidemiologic Studies Depression Scale (CES-D), and Pain Catastrophizing Scale (PCS) enhances the reliability and comparability of the findings with existing literature. Third, the inclusion of a relatively robust clinical sample of 67 patients with CLBP strengthens the statistical validity of the analyses and increases confidence in the observed associations between central sensitization and neuropsychological variables. Finally, the stratification of patients based on a validated CSI cut-off allows for clinically meaningful group comparisons, supporting the translational relevance of the results for clinical assessment and personalized intervention planning.

## 6. Limitations

Despite these strengths, several limitations should be acknowledged. The cross-sectional design of the study precludes any causal inference regarding the relationships between central sensitization, cognitive performance, emotional distress, and pain-related cognitions. Longitudinal studies are needed to clarify the temporal dynamics and directionality of these associations. Although the sample size is adequate, participants were recruited from a single clinical center, which may limit the generalizability of the findings to broader CLBP populations. Additionally, cognitive functioning was assessed using a global screening tool (MoCA), which, while clinically practical, does not allow for a fine-grained analysis of specific cognitive domains. Another limitation is the reliance on self-report questionnaires for the assessment of central sensitization, depressive symptoms, and pain catastrophizing, which may be subject to response bias. In addition, potential confounding variables such as drug therapy, additional comorbidities, and lifestyle, were not fully controlled for. In particular, data on physical activity levels and the use of drugs that could influence central sensitization, such as antidepressants, anxiolytics, and analgesics, were not analyzed in this study. Finally, the study did not include objective neurophysiological measures of pain processing, limiting the ability to directly link subjective reports of sensitization with underlying neural mechanisms.

## 7. Future Perspectives

Future research should aim to integrate subjective clinical assessments with objective neurophysiological measures to achieve a more comprehensive characterization of pain processing in CLBP. Electrophysiological biomarkers such as cognitive event-related potentials (e.g., P300), which provide objective indices of cognitive processing and attentional allocation, and Laser Evoked Potentials (LEPs), which assess nociceptive system functioning and central pain processing, represent promising complementary approaches. Integrating these techniques with psychometric and neuropsychological assessments could strengthen the understanding of neurophysiological mechanisms underlying central sensitization and cognitive vulnerability in chronic low back pain. In particular, LEPs may help elucidate the neural correlates of altered pain processing and clarify their relationship with reduced cognitive performance and emotional dysregulation. Moreover, longitudinal studies incorporating these measures may improve patient stratification, contribute to the development of more targeted multimodal interventions, and help identify biomarkers of treatment response and disease progression, ultimately supporting more effective clinical decision-making in chronic low back pain.

## 8. Conclusions

Central sensitization in chronic low back pain identifies a subgroup of patients with reduced cognitive performance, more severe depressive symptoms, and increased catastrophic thinking. These findings support central sensitization as an independent marker of multidimensional clinical vulnerability. Early identification may facilitate personalized multimodal interventions aimed at improving cognitive, emotional, and overall clinical outcomes.

## Figures and Tables

**Table 1:** Detailed description of the instruments.

Domain	Instrument	Type	Structure
**Central sensitization**	Central Sensitization Inventory (CSI) [20]	Psychometric	25-item Likert-type questionnaire
**Cognitive function**	Montreal Cognitive Assessment (MoCA) [21]	Neuropsychological	Multi-domain cognitive screening
**Depressive symptoms**	Center for Epidemiologic Studies Depression Scale (CES-D) [22]	Psychometric	20-item self-report questionnaire
**Pain catastrophizing**	Pain Catastrophizing Scale (PCS) [23]	Psychometric	13-item scale (rumination, magnification, helplessness)

**Table 2:** Participant Characteristics and Unadjusted Between-Group Comparisons.

Variable	WCS (*n* = 35)	WoCS (*n* = 32)	*p*-Value
Age (years), median (IQR)	58 (51–61)	50 (35–54)	**0.001** ^†^
Female, *n* (%)	14 (40.0%)	23 (71.9%)	**0.018** ^‡^
CSI, Mean ± SD	21.9 ± 10.2	57.5 ± 11.9	**<0.001**
PCS, Mean ± SD	16.0 ± 11.9	31.8 ± 14.2	**<0.001**
MoCA, Mean ± SD	26.1 ± 2.1	23.6 ± 3.0	**<0.001**
CES-D, Mean ± SD	11.7 ± 7.2	26.3 ± 10.4	**<0.001**

^†^ Mann–Whitney U test; ^‡^ Chi-square test; All other comparisons: Welch’s independent-samples *t*-test.

**Table 3:** Multivariable linear regression analyses examining the association between central sensitization and clinical outcomes (adjusted for age and gender).

Variable	B (95% CI)	β	*p*-Value
MoCA	2.41 (1.36 to 3.46)	0.41	<0.001
CES-D	−14.62 (−18.7 to −10.5)	−0.64	<0.001
PCS total score	−15.24 (−19.8 to −10.6)	−0.52	<0.001
PCS helplessness	−7.31 (−9.6 to −5.0)	−0.49	<0.001
PCS rumination	−5.63 (−7.9 to −3.3)	−0.42	<0.001
PCS magnification	−2.71 (−3.7 to −1.7)	−0.38	<0.001

## Data Availability

The data presented in this study are not publicly available due to privacy and ethical restrictions, as they contain sensitive clinical information that could compromise participant confidentiality.
